# Anterior Cervical Discectomy and Fusion (ACDF): Comparison Between Zero Profile Implants and Anterior Cervical Plate and Spacer

**DOI:** 10.7759/cureus.573

**Published:** 2016-04-17

**Authors:** Marjan Alimi, Innocent Njoku, Christoph P Hofstetter, Apostolos J Tsiouris, Kartik Kesavabhotla, John Boockvar, Rodrigo Navarro-Ramirez, Roger Härtl

**Affiliations:** 1 Department of Neurosurgery, Weill Cornell Medical College, New York Presbyterian Hospital, New York; 2 Radiology, Division of Neuroradiology, Weill Cornell Medical College, New York Presbyterian Hospital, New York; 3 Department of Neurosurgery, Lenox Hill Hospital, New York

**Keywords:** anterior cervical discectomy and fusion, acdf, anterior plate, clinical outcome, dysphagia, radiographic, zero-profile

## Abstract

Introduction: Interposition grafts combined with anterior plating currently remain the gold standard for anterior cervical discectomy and fusion. The use of anterior plates increases fusion rates but may be associated with higher rates of postoperative dysphagia. The aim of the current study was to determine the clinical and radiological outcomes following anterior cervical discectomy and fusion (ACDF) using zero-profile anchored spacers versus standard interposition grafts with anterior plating.

Methods: This was a retrospective case series. A total of 53 male and 51 female consecutive patients (164 total operated levels) who underwent ACDF between 2007 and 2011 were included. The mean clinical follow-up was 15.7 ± 1.2 (SEM) months for patients with zero-profile implants and 14.8 ± 2.1 months for patients with conventional ACDF with anterior plating. Patient demographics, operative details, clinical outcomes, complications, and radiographic imaging were reviewed. Dysphagia was determined using the Bazaz criteria.

Results: Clinical outcome scores improved in both groups as measured by the modified Japanese Orthopedic Association and Nurick scores. Zero-profile constructs gave rise to significantly less prevertebral soft tissue swelling compared to constructs with anterior plates postoperatively (15.74 ± 0.52 as compared to 20.48 ± 0.85 mm, p < 0.001) and at the latest follow-up (10.88 ± 0.39 mm vs. 13.72 ± 0.67 mm, p < 0.001). There was a significant difference in the incidence of dysphagia at the latest follow-up (1.5% vs. 20%, p=0.001, zero-profile vs. anterior plate, respectively).

Conclusion: Zero-profile implants lead to functional outcomes similar to standard anterior plate constructs. Avoiding the use of an anterior locking plate may decrease the risk of persistent postoperative dysphagia.

## Introduction

Anterior cervical discectomy and fusion (ACDF) is a well-established technique for treatment of cervical myelopathy and radiculopathy. The technique aims at establishing neural decompression and providing segmental stability at the symptomatic cervical level [1]. Since its initial description nearly 50 years ago, the technique has undergone extensive modifications [2-3]. The current standard method combines an anterior locking plate with either a synthetic, allograft, or metallic interposition graft [3-9]. While the addition of an anterior plate enhances the biomechanical stability of the construct and leads to a higher fusion rate, it has also been associated with prevertebral soft tissue injury and dysphagia [10-14].

Several factors are assumed to have a role in the increased rates of dysphagia following ACDF with anterior plating, such as retraction, direct impingement of the esophagus, and irritation of surrounding soft tissue [15-18].

A zero-profile plate may exert less of a mass effect on the esophagus. It has been approved by the FDA for anterior intervertebral screw fixation of the cervical spine at levels C2-T1. Recently, several studies have demonstrated the utility of a zero-profile integrated plate and spacer and documented its clinical as well as radiographic outcome measures [19-20]. ACDF with plating has been found by many studies to be associated with a high rate of postoperative dysphagia, up to a clinical incidence of 51% in some studies [11-12, 16, 21]. A previous study showed that zero-profile anchored spacers can lead to similar clinical and radiographical outcomes as ACDF with plating, yet carry a lower risk for persistent dysphagia [14].

In the current study, we compared zero-profile devices to conventional ACDF with an anterior plate, focusing on the rate of persistent dysphagia and prevertebral soft-tissue swelling. 

## Materials and methods

### Patient population

We conducted a retrospective analysis of a prospectively collected database, assessing patients who underwent an ACDF between October 2007 and October 2011 at a single center.

A total of 104 patients with 164 operated levels were included. Our current series encompasses one-third of our patients receiving the Zero-P implant (Synthes®, West Chester, PA), another third receiving the ROI-C implant (LDR-Spine, Austin, TX), and the final third receiving a DePuy carbon-fiber cage (BENGAL®, DePuy Synthes) combined with an anterior plate (SKYLINE®, DePuy Synthes) (Table 1).

**Table 1:** Implant Characteristics and Versatility

	Zero-Profile Device		Anterior Plate
Product	Zero-P	ROI-C (LDR)	Skyline
Material	PEEK with integrated titanium plate	PEEK	Titanium plate
Depth x Width (mm)	13.5 x 17.5	12 x 14; 12 x 15.5; 14 x 14; 14 x 15.5; 14 x 17	N/A
Height (mm)	5, 6, 7, 8, 9, 10, 11, 12	4.5, 5, 5.1, 5.5, 6, 6.5, 7, 7.5, 8, 8.5	12 - 102
Anterior Thickness (mm)	0	0	2.5
Sagittal Profile	Convex, parallel, lordotic	Convex	Lordotic
Anchoring Device	4 screw construct, self-tapping, 3.0 mm anchoring device	2 screw construct, self-drilling, 3.5 mm diameter	4-12 screw construct, self-tapping, self-drilling
Screw Length (mm)	12, 14, 16	8, 10, 12, 24	10, 12, 13, 14, 15, 16, 17, 18, 20, 22, 24, 26
Screw Angulation	Fixed only	Parallel insertion	5º - 20º angulation
Blocking Mechanism	Screw threads into the plate	Self-locking clips	Screw-threaded interplate

The first and second third were grouped together as patients receiving zero-profile implants and were compared to the final third, who received anterior plates.

Although patients were not randomized, statistical tests were performed to assess whether the demographic and/or peri-operative characteristics of the patients were significantly different between the two groups. All patients had symptomatic degenerative cervical disc disease or disc herniation between the levels of C3/4 to C7/T1. Epidemiological data, the location of pathology, levels of fixation, surgery time, estimated blood loss, the length of hospital stay, complications, and follow-up data were collected. Institutional review board approval was obtained for this project.

### Surgical technique

In this procedure, patients were intubated via general endotracheal techniques. We utilized a Smith-Robinson approach to reach the diseased level(s) [3]. Patients were positioned supine on the operating table. A horizontal curvilinear incision was made from midline to the anterior aspect of the sternocleidomastoid muscle. Soft tissue was dissected and the carotid artery was mobilized laterally while the trachea and esophagus were mobilized medially. Once the prevertebral space was accessed, the medial longus colli muscle was dissected off the vertebral bodies. Distraction pins were placed under fluoroscopic guidance and osteophytes were removed with a high-speed drill under the operating room microscope. Discectomies were performed using straight curettes and pituitary rongeurs. The posterior longitudinal ligament was resected using Kerrison rongeurs.

The wound was irrigated and closed in standard fashion. Patients received standard postoperative care and appropriate analgesic medication in addition to gastric ulcer and deep venous thrombosis prophylaxis. Patients were placed in a collar postoperatively only if they were smokers.

### Clinical evaluation

Clinical outcome scores were collected preoperatively, immediately postoperatively, and at the latest follow-up. Neurological impairment was assessed by the Nurick [22] and modified Japanese Orthopedic Association scores (JOA) [23-24]. Dysphagia was recorded and graded according to the Bazaz scoring system [12]. A numerical score ranging from 0 (no episodes of swallowing difficulty) to 3 (severe difficulty with the majority of the food) was noted for each patient. Intraoperative and postoperative complications were recorded.

### Radiographic evaluation

Anteroposterior (AP) and lateral cervical radiographs were used to evaluate the cervical prevertebral soft tissue thickness, as well as hardware failure and/or instability immediately postoperatively and at the latest follow-up. The prevertebral soft tissue thickness was measured in the mid-portion of the fusion construct. Measurements were recorded to the nearest half millimeter. The occurrence of bony fusion was assessed on the AP and lateral cervical spine x-rays six months following surgery. In patients with an available CT scan, the fusion was assessed using the latter imaging. Trabecular bridging across the bone-graft interface and absence of radiolucent gaps between the graft and vertebral endplate were the radiographic criteria used to assess fusion.

### Statistical analysis

Continuous variables are shown either as mean ± standard error from means or as median (min; max). Percentages were calculated for categorical variables. Differences between the two compared groups in the categorical variables were assessed using a Chi-square test. Differences between continuous variables were tested using a Student’s T-test. All analyses were performed using appropriate statistical software (SPSS Version 18.0.0.1, SPSS Inc., Chicago, IL).

## Results

### Demographic and perioperative data

**Figure 1:**
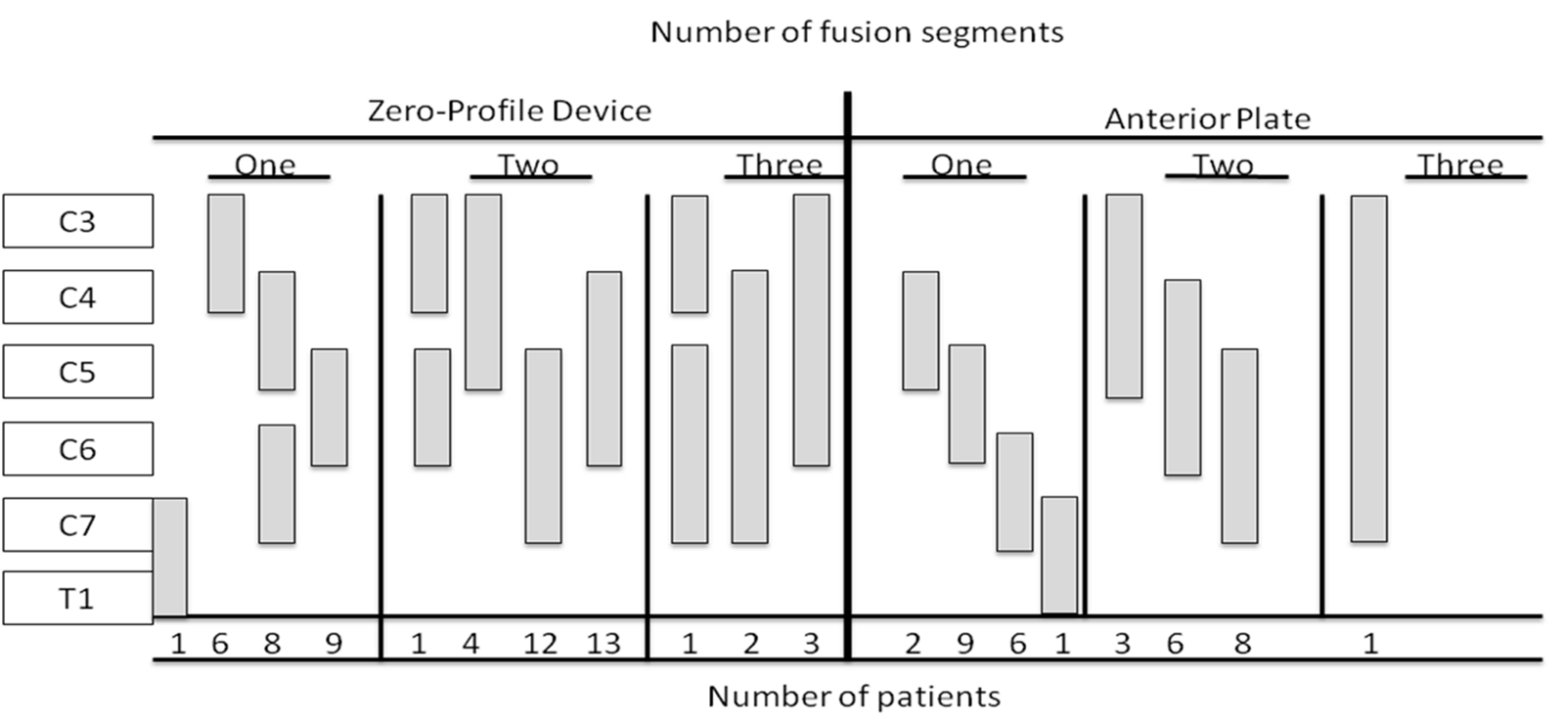
Illustration of Fused Vertebral Segments as a Function of the Number of Operated Patients

One hundred and four patients (34 Zero-P, 35 ROI-C, and 35 anterior plates) were operated on with a combined total of 164 levels (110 levels with a zero-profile device and 54 levels with an anterior plate) (Figure 1).

Our cohort consisted of 53 male and 51 female patients. The average age of the cohort was 55.9 ± 1.20. Patients who received a zero-profile device were significantly older than patients who received a standard construct with an anterior plate (58.2 ± 1.45 years vs. 51.5 ± 1.95, respectively, P = 0.007). The gender distribution, mean BMI, and the severity of the preoperative neurological impairment were similar between the two groups. Also, there were no statistically significant differences between the two groups in the number of operated levels, estimated blood loss, and/or duration of the follow-up (Tables 2-3).

**Table 2:** Patient Characteristics *P values < 0.05 are considered to be statistically significant **Denotes the difference in gender distribution (male - female) ¹Mean ± SE

*Patient Characteristics*		All Cases (104 pts)	ROI-C & Zero-P (69 pts)	Anterior Plate (35 pts)	P value
Age at surgery (years) ¹		55.9 ± 1.20	58.2 ± 1.45	51.5 ± 1.95	0.007*
Gender	Male	53 (51%)	35 (50.7%)	18 (51.4%)	0.946**
	Female	51 (49%)	34 (49.3%)	17 (48.6%)	
BMI (kg/m²) ¹		28.1 ± 0.66	28.0 ± 0.76	28.2 ± 1.36	0.924*

**Table 3:** Surgical Details and Operated Levels *P values < 0.05 are considered to be statistically significant ¹Estimated Blood Loss (EBL); Median (min; max) ^2 ^Mean ± SE

Surgical Details		All Cases (104 pts, 164 Levels)	ROI-C & Zero-P (69 pts, 110 Levels)	Anterior Plate (35 pts, 54 Levels)	P value
Number of levels / patient	1 level	49 (47.1%)	32 (46.4%)	17 (48.6%)	
	2 levels	50 (48.1%)	33 (47.8%)	17 (48.6%)	0.935
	3 levels	5 (4.8%)	4 (5.8%)	1 (2.9%)	
Level of surgery	C3/C4	19 (11.6%)	15 (13.6%)	4 (7.4%)	
	C4/C5	42 (25.6%)	30 (27.2%)	12 (22.3%)	
	C5/C6	64 (39.0%)	41 (37.2%)	23 (42.6%)	
	C6/C7	36 (22.0%)	22 (20.0%)	14 (25.9%)	
	C7/T1	3 (1.8%)	2 (1.8%)	1 (1.8%)	
Estimated Blood Loss (EBL)¹		50 (0; 600)	50 (0; 600)	50 (20; 500)	0.109
Follow-up duration^2^		15.4 ± 1.08	15.7 ± 1.23	14.8 ± 2.13	0.688

### Clinical results

Clinical outcomes were assessed using the modified JOA and the Nurick scores (Table 4).

**Table 4:** Clinical Outcome in All Patients and in Each Group *P values < 0.05 are considered to be statistically significant ¹ Mean ± SE

Clinical Outcome (In Each Group)^1^	Preoperative	Latest Follow-up	P value
All cases			
Nurick	0.96 ± 0.11	0.57 ± 0.07	< 0.001*
JOA	13.62 ± 0.17	15.51 ± 0.15	< 0.001*
ROI-C & Zero-P (69 patients, 110 levels)			
Nurick	1.19 ± 0.14	0.65 ± 0.09	< 0.001*
JOA	13.54 ± 0.24	15.57 ± 0.20	< 0.001*
Anterior Plate (35 patients, 54 levels)			
Nurick	0.51 ± 0.12	0.43 ± 0.10	0.083
JOA	13.77 ± 0.18	15.40 ± 0.23	< 0.001*

Patients who underwent implantation of the zero-profile device had functional outcomes comparable to the patients who received conventional constructs with anterior plating (Table 5).

**Table 5:** Comparison of the Clinical Outcome Between Groups *P values < 0.05 are considered to be statistically significant ¹Mean ± SE

Clinical Outcome (Comparison Between Groups)^ 1^	All Cases (104 pts)	ROI-C & Zero-P (69 pts)	Anterior Plate (35 pts)	P value (Intergroup Difference)
Preoperative Nurick	0.96 ± 0.11	1.19 ± 0.14	0.51 ± 0.12	0.002 *
Last Follow-up Nurick	0.57 ± 0.07	0.65 ± 0.09	0.43 ± 0.10	0.147
Nurick Improvement	0.38 ± 0.09	0.53 ± 0.13	0.09 ± 0.04	0.023 *
Preoperative JOA	13.62 ± 0.17	13.54 ± 0.24	13.77 ± 0.18	0.540
Last Follow-up JOA	15.51 ± 0.15	15.57 ± 0.20	15.40 ± 0.23	0.605
JOA Improvement	1.88 ± 0.14	2.00 ± 0.19	1.63 ± 0.21	0.238

Dysphagia improved significantly in all patients from the postoperative time to the latest follow-up; this improvement could also be observed separately in each group (Table 6).

**Table 6:** Dysphagia in All Patients and in Each Group *P values < 0.05 are considered to be statistically significant ¹ Mean ± SE

Dysphagia (In Each Group) ^1^	Immediate Postoperative	Latest Follow-up	P value
All cases			
Dysphagia score	0.70 ± 0.09	0.14 ± 0.03	< 0.001*
ROI-C & Zero-P (69 patients, 110 levels)			
Dysphagia score	0.85 ± 0.12	0.10 ± 0.04	< 0.001*
Anterior Plate (35 patients, 54 levels)			
Dysphagia score	0.40 ± 0.08	0.20 ± 0.06	< 0.006 *

Although the average immediate postoperative dysphagia score was higher in the zero-profile group compared to the anterior plate group (0.85 ± 0.12 vs. 0.40 ± 0.08, respectively; p=0.018), the extent of improvement at the latest follow-up time was significantly higher in zero-profile group as well, compared to the anterior plate group (0.75 ± 0.11 vs. 0.20 ± 0.69, respectively; p=0.002). Correspondingly, the dysphagia score at the latest follow-up was not significantly different between the two groups (Table 7).

**Table 7:** Comparison of Dysphagia Between Groups *P values <0.05 are considered to be statistically significant ¹ Mean ± SE

Dysphagia (Comparison Between Groups)	All Cases (104 pts)	ROI-C & Zero-P (69 pts)	Anterior Plate (35 pts)	P value (Intergroup Difference)
Immediate Postoperative Dysphagia Score ^1^	0.70 ± 0.09	0.85 ± 0.12	0.40 ± 0.08	0.018 *
Latest Follow-up Dysphagia Score ^1^	0.14 ± 0.03	0.10 ± 0.04	0.20 ± 0.06	0.211
Dysphagia Score Improvement ^1^	0.56 ± 0.08	0.75 ± 0.11	0.20 ± 0.69	0.002 *
Immediate Postoperative Dysphagia Incidence	33 (31.7%)	19 (27.9%)	14 (40.0%)	0.214
Latest Follow-up Dysphagia Incidence	8 (7.7%)	1 (1.5%)	7 (20.0%)	0.001 *
Dysphagia Incidence Improvement	25 (24%)	18 (26.1%)	7 (20.0%)	0.468

In addition to the Nurick score, patients were assessed for the presence or absence of dysphagia. In the postoperative period, 27.9% of patients with the zero-profile implant experienced transient dysphagia as compared to 40% of the patients in the anterior plate group. However, at the latest follow-up, 1.5% of patients in the zero-profile group continued experiencing dysphagia, compared to 20% in the anterior plate group (p = 0.001).

### Radiographic results

Prevertebral soft tissue thickness significantly decreased from the postoperative time to the latest follow-up in all patients as well as in each group separately (Table 8).

**Table 8:** Prevertebral Soft Tissue Thickness in All Patients and in Each Group *P values < 0.05 are considered to be statistically significant ¹ Mean ± SE

Prevertebral Soft Tissue Thickness (In Each Group) ^1^	Immediate Postoperative	Latest Follow-up	P value
All cases			
Tissue thickness	17.30 ± 0.50	11.81 ± 0.36	< 0.001*
ROI-C & Zero-P (69 patients, 110 levels)			
Tissue thickness	15.74 ± 0.52	10.88 ± 0.39	< 0.001*
Anterior Plate (35 patients, 54 levels)			
Tissue thickness	20.48 ± 0.85	13.72 ± 0.67	< 0.001*

Nonetheless, the postoperative prevertebral soft tissue thickness was significantly lower in patients with the zero-profile implants compared to those with the anterior plate (15.74 ± 0.52 mm vs. 20.48 ± 0.85 mm, respectively; p < 0.001) (Table 9).

**Table 9:** Comparison of Prevertebral Soft Tissue Thickness Between Groups * P values < 0.05 are considered to be statistically significant ¹ Mean ± SE

Prevertebral Soft Tissue Thickness (Comparison Between Groups) ^1^	All Cases (104 pts)	ROI-C & Zero-P (69 pts)	Anterior Plate (35 pts)	P value (Intergroup Difference)
Immediate postoperative tissue thickness	17.30 ± 0.50	15.74 ± 0.52	20.48 ± 0.85	< 0.0001 *
Latest follow-up tissue thickness	11.81 ± 0.36	10.88 ± 0.39	13.72 ± 0.67	< 0.0001 *
Tissue thickness reduction	5.41 ± 0.42	4.90 ± 0.47	6.52 ± 0.84	0.073

At the latest follow-up, the prevertebral soft tissue thickness remained significantly lower in the zero-profile group as compared to the anterior plate group (10.88 ± 0.39 mm vs. 13.72 ± 0.67 mm, p < 0.001). The radiographic fusion rates for patients undergoing an ACDF with the zero-profile implant and with anterior plate were 92.1% and 96%, respectively. The difference in the fusion rate between the two groups was not statistically significant (p=0.501). In addition, there were no statistically significant differences in the fusion rates for single level, two level, and three level surgeries.

## Discussion

ACDF constitutes a well-established treatment modality for cervical myelopathy and radiculopathy. In our current study, we demonstrated that a zero-profile device allows for similar clinical and radiographic outcomes compared to a conventional construct with an anterior plate, while reducing the rate of postoperative dysphagia.

Overall, it seems that dysphagia is still a major concern for this procedure [11-12, 16, 21]. Hofstetter, et al. showed that zero-profile anchored spacers can result in a lower dysphagic incidence when compared to historical data on anterior plate surgery [14]. Similarly, our study showed lower rates of postoperative dysphagia with zero profile devices, regardless of the manufacturing company, compared to an anterior locking plate. The chronic dysphagia rate in our cohort of patients utilizing a zero-profile device was 1.5%, which was lower than the rates reported in the literature for studies utilizing traditional anterior plate and spacer constructs [11-12, 16, 21, 25-26].

### Dysphagia

Chronic dysphagia is a well-known complication following ACDF with an anterior plate [12, 16-17, 27]. The pathophysiology of dysphagia after ACDF is not well understood but it appears to be caused by multiple factors, such as retraction during implantation, irritation of tissue, and mass effect on the esophagus [17, 28].

Several factors have been proposed to contribute to increased rates of dysphagia following ACDF with anterior plating, among which are additional retractions required for plate implantation, direct impingement of the esophagus by the plate as well as irritation of surrounding soft tissue [15-16].

One plausible explanation underlying the increased risk of dysphagia with anterior plating may be due to additional esophageal retraction necessary for placement and implantation of the anterior plate. Pressure applied by the retractor blade onto the pharynx/esophagus can cause local ischemic damage inside the esophageal wall. Additionally, it is conceivable to attribute such complications to soft tissue edema, postoperative hematoma, or esophageal irritation [17]. Direct esophageal contact between the device and soft tissue can also add to irritation leading to soft tissue edema of the esophageal wall [18].

It has been suggested that plate design may play a substantial role in the development of chronic dysphagia. The thickness of the cervical plate and its position directly posterior to the esophagus may contribute to this complication by causing soft tissue irritation [12, 27, 29]. A study done by Mobbs, et al. showed significantly higher rates of dysphagia in patients who received anterior plates as compared to constructs without anterior plating [29]. In a two-year prospective longitudinal follow-up study with 156 patients, Lee, et al. found that utilization of smaller and smoother profile plates was associated with decreased incidence of dysphagia compared to the implantation of slightly larger and less smooth plates [27].

### Design of the implant

Overall, there are several advantages to the utilization of zero-profile devices. Zero-P device has plates that are integrated within the intervertebral disc site. This reduces impingement of prevertebral structures, such as the esophagus. The small titanium plate comes preassembled with an interbody spacer and provides a rigid locking interface for anchorage. The entire implant is contained within the disc space and eliminates the need for instrument protrusion outside the intervertebral space as in the case anterior cervical plate devices. Screws or anchorage blades are placed within the plate at a medial or lateral angle for fixation. A biomechanical study of low profile stand-alone cervical anterior cage device on 24 human cadaveric cervical spines, conducted by Scholz, et al., demonstrated similar mechanical stability to the traditional ACDF techniques using a standard anterior plate and cage designs [26].

ROI-C implant’s characteristics are also similar to those of Zero-P device in many aspects (Table 1).

Being available in a variety of heights and footprints to match the patient’s anatomy, ROI-C implants accommodate integrated, self-guided, self-locking plating designed to provide stability without a need for additional instrumentation protruding anterior of the vertebral bodies. The self-guided, curved plates are delivered in the plane of the disc through a direct anterior approach so that the surgery can be achieved with less exposure than may be required to implant a traditional cervical plate, or even contemporary stand-alone systems with screws that must be inserted at oblique and challenging angles.

Moreover, due to the implant design, there are significant reductions in operative time with fewer surgical steps as compared to procedures done with an anterior locking plate [30].

The findings of the current study suggest that in comparison to the anterior plate, utilization of the zero-profile device (regardless of the type) is associated with a reduced risk of persistent dysphagia while resulting in a comparable and favorable clinical outcome.

In addition, prevertebral soft tissue swelling is a common observation after ACDF. The severity of the prevertebral soft tissue edema, however, may in part be impacted by the type of instrumentation. In the present study, patients undergoing ACDF with a zero-profile device developed significantly less prevertebral soft tissue swelling as compared to those with an anterior plate. This trend was observed both at short-term (< 3 months) and long-term (> 3 months) follow-up.

### Study limitations

Several limitations exist in the design of the current study. We conducted a retrospective study of 104 patients comparing the clinical and radiographic outcome of the zero-profile device with the standard anterior plating. Patients were not randomized into different groups; therefore, a potential for selection bias exists. The retrospective nature of the study and the relatively low number of cases limits the statistical power of the study and necessitates interpretation of the results with precaution.

Nevertheless, statistical tests were performed to assess whether there were statistically significant differences between the two groups in the demographic and/or perioperative characteristics in which there were none except for a slight difference in the average age. The other limitation of the study was the lack of the preoperative soft tissue thickness measurement. As a result, the soft tissue thickness had been measured and compared between the two groups at the postoperative and the latest follow-up time points.

To attribute postoperative dysphagia in ACDF to the anterior plating system, double-blinded randomized controlled studies are needed.

## Conclusions

Zero-profile devices are associated with similar clinical and functional outcomes as conventional constructs with anterior plates; nonetheless, the prevertebral soft tissue thickness and persistent dysphagia rates are lower with zero-profile implants. Further studies are required to delineate the pathophysiological mechanisms underlying dysphagia following ACDF.
